# Qualitative Assessment of the Application of a Discrete Choice Experiment With Community Health Workers in Uganda: Aligning Incentives With Preferences

**DOI:** 10.9745/GHSP-D-16-00070

**Published:** 2016-12-23

**Authors:** Aurélie Brunie, Mario Chen, Angela Akol

**Affiliations:** a FHI 360, Washington, DC, USA.; b FHI 360, Durham, NC, USA.; c FHI 360, Kampala, Uganda.

## Abstract

**Background:**

Maximizing the benefits of community health worker (CHW) programs requires strategies for improving motivation, performance, and retention. Discrete choice experiments (DCE) are increasingly used to inform policy response to health workforce shortages in rural areas, and may be of value in the context of CHW programs. Participants are presented with pairs of hypothetical jobs that are described by job attributes with varying levels and are asked what their preferred job is within each pair. Responses are then analyzed quantitatively to obtain information on what attributes are important to participants. We conducted a qualitative assessment to examine the appropriateness and validity of applying a DCE to a new population of CHWs with lower literacy.

**Methods:**

In 2011, we conducted a mixed-method study with CHWs in Uganda, consisting of 183 surveys and 43 in-depth interviews (IDIs). The DCE was administered to both survey and IDI participants. This article reports on the qualitative assessment of the implementation of the DCE. We compare DCE responses between survey and IDI participants to determine whether administering the DCE in a qualitative (IDI) context altered responses. We then present additional information collected on CHWs' decision-making processes and their experiences with the DCE in the IDIs.

**Results:**

Choices made by IDI participants were consistent with the choices made by survey participants. In-depth exploration of CHWs' observations in answering the DCE suggest that, overall, CHWs comprehended the DCE exercise and made reasoned choices. However, the data revealed some level of cognitive difficulty and highlighted some design and implementation challenges that are important to consider, particularly when applying a DCE to populations with lower literacy. These include the need to keep the number of attributes small; to choose levels that are realistic yet show sufficient range; and to clearly define attributes and their levels.

**Conclusion:**

DCEs can be an appropriate approach with CHWs but require careful design and implementation.

## Abstract (precis)

Conducting a discrete choice experiment (DCE) with CHWs via survey versus interviews gave similar findings: the most appealing attributes for these CHWs were a bicycle, transportation refund, and mobile phone. To promote meaningful and valid results, particularly when applying DCEs to lower-literacy populations such as CHWs, DCEs should (1) use a small number of job attributes to facilitate comprehension, (2) choose attribute levels (e.g., mobile phone vs. no mobile phone) that are realistic yet show sufficient range, and (3) clearly define attributes and their levels.

## INTRODUCTION

Community health workers (CHWs) bring health services to the rural poor who often have little or no access to the primary health care system. CHWs typically are lay community members who receive a limited amount of training to carry out one or more basic health functions in their village. The services provided vary across contexts as does compensation, with some CHWs working as volunteers and others receiving payment for their work. Keeping CHWs motivated to perform well and stay on the job is critical to the cost-effectiveness, impact, and sustainability of CHW programs,^1^^–^^4^ yet it is challenging, particularly in volunteer programs. An array of non-financial incentives, such as T-shirts or bags, as well as including programmatic elements such as training or supervision, have been shown to have motivational value and to affect performance and retention outcomes.^1^^–^^3^^,^^5^ There is no magic formula, however, to ensure that CHWs stay motivated and productive, and finding the right mix of incentives is a complex issue that depends on the specific context.^2^^,^^6^

In Uganda, both public- and private-sector programs have used CHWs to deliver information and services, including family planning, since the 1980s. In an effort to streamline these efforts and systematically empower and mobilize communities for health, the government rolled out a nationwide Village Health Team strategy beginning in 2004, whereby teams of volunteers provide the platform for all community-based health programming. Although the Ministry of Health defined a set of minimum, non-financial incentives for the Village Health Team model in 2009, identifying incentive packages that appropriately motivate volunteers remains the subject of many discussions among stakeholders, including the ministry and its implementing partners.

In the past 5 years, the use of discrete choice experiments (DCEs) has gained prominence as a tool for identifying strategies to make rural jobs more attractive to health workers in resource-limited settings,^7^^–^^13^ including in Uganda.^14^ DCE is an analytic technique for eliciting stated preferences that involves presenting respondents with pairs of hypothetical scenarios (e.g., job postings) described in terms of bundles of attributes (e.g., location, salary, or equipment) that vary in their levels (e.g., urban vs. rural posting). Respondents select their preferred scenario within each pair, and response data are analyzed to estimate the influence of each attribute on their choice. The approach mimics real-life decisions because it forces participants to consider trade-offs among wanted attributes when choosing between two scenarios. DCEs can be used in the absence of empirical choice data and permit the inclusion of various incentive options that are not currently being implemented.^10^^,^^15^^,^^16^ Although DCE has been applied with low-literacy populations on other health systems topics (such as rural women's preferences for place of delivery in Tanzania),^17^ to our knowledge, completed research involving its use to reveal incentive preferences has been conducted almost exclusively with professional health workers, including doctors, nurses, and medical students. At the time this study was conducted, DCE research had not been extended to lay workers such as CHWs; since then, findings from only one other exploratory DCE with CHWs have been reported.^18^

Discrete choice experiments require participants to consider trade-offs between job attributes when choosing between two job scenarios.

We implemented a DCE as part of a mixed-method study on the factors affecting the motivation and performance of CHWs in 3 family planning programs in Uganda. Full study findings that include the main quantitative DCE results are reported elsewhere.^19^ Here, we present additional results from the concurrent administration of the DCE as part of the qualitative component of the study. Our objective was to obtain data that would unveil CHWs' reasoning and experiences with the exercise in order to validate the quantitative findings of the DCE in our context. Specifically, we wanted to examine whether CHWs fully considered all attributes when going through choice tasks, as opposed to choosing between two jobs based on a single or subset of attributes, and whether they made reasoned and deliberate decisions. We were also interested in assessing how comprehensible and cognitively demanding the exercise was.

We conducted a qualitative study to unveil CHWs' reasoning and experiences with a discrete choice experiment.

## METHODS

The design and DCE methods of this study, which received ethical approval from the Uganda National Council for Science and Technology and FHI 360's Protection of Human Subjects Committee, were fully described in a previous paper.^19^ Briefly, we conducted a cross-sectional study in 2011 with CHWs from 3 family planning programs covering 7 of Uganda's 112 districts: one program was operated in the public sector, one was supported by an NGO, and one had recently transitioned from an NGO to the public sector. We selected CHWs who had at least one year of experience distributing contraceptives (including condoms, pills, and possibly, but not necessarily, injectables) and had attended their last supervisory meeting or had a documented excuse for missing it. These criteria were meant to focus the study on CHWs who were currently active and had volunteered long enough to have experienced all the realities of their work, allowing us to explore the full range of factors affecting motivation. The main study included both a survey and in-depth interviews (IDIs) with CHWs. In the largest district for each of the 3 family planning programs, CHWs were randomly selected to participate in either the survey or an IDI; everywhere else, all CHWs participated in the survey. A total of 183 CHWs completed the survey and 43 completed an IDI, corresponding to a combined response rate of 91% of all CHWs approached for recruitment into these data collection components.

To inform the selection of job attributes and the levels of these attributes for the DCE, we reviewed the relevant literature and convened a meeting with stakeholders working with CHWs in Uganda. Based on the results of this consultation, we identified a final set of 5 locally relevant and realistic attributes and between 2 and 3 levels for each (Table 1). We used the %ChoiceEff macro in SAS (SAS, Cary, NC) to generate an optimal fractional factorial design through the selection of 24 of the 48 possible combinations of attributes and levels, each corresponding to a job profile; the 24 job profiles were organized in 12 pairs.^20^ Each job is thus characterized by the levels specified for each of the 5 attributes and includes the higher level for some attributes and the lower level for others.

**TABLE 1.** Discrete Choice Experiment Attributes and Levels, Uganda, 2011

Attributes	Definition	Level
Supervision	Frequency and location of supervisory meetings	Monthly CHW meetings at health center Same as (1) + quarterly visit by health center staff in the community
Training	Frequency and duration of initial and refresher training	5-day initial training and 3-day supervised practicum at health center Same as (1) + 3-day refresher training once a year
Transportation refund	Transportation refund received for each supervisory meeting attended	5,000 UGX per meeting 10,000 UGX per meeting
Start-up package	Items received upon joining the CHW program (one-time)	CHW kit with gumboots, raincoat, job aids, and stationery CHW kit + T-shirt + badge CHW kit + T-shirt + badge + bicycle
Communication	One-time provision of a mobile phone to communicate with program staff	No mobile phone Mobile phone without airtime

Abbreviations: CHW, community health worker; UGX, Ugandan shilling.

The DCE was part of the survey questionnaire. Since we considered that this was a novel application of this approach with CHWs, we also administered the DCE questions to IDI participants, along with additional probing to elicit the rationale behind their decisions and to collect their impressions of the exercise. To keep the task presented to each respondent manageable in the context of the broader survey or IDI, we partitioned the profiles into 4 blocks of 3 pairs of jobs each. Table 2 shows an illustrative pair. CHWs in both the survey and IDI groups were randomly allocated to receive one of the 4 blocks. To facilitate understanding and ensure consistent implementation, trained research assistants read from a script to explain the DCE exercise, then successively presented each CHW with cards describing 3 pairs of jobs in their assigned block and read aloud the job descriptions to the participant. Cards were translated and interviews were conducted in Luganda, Lusoga, or Samia. Research assistants were oriented to the DCE methodology through a training session; supervised role play and a field pretest provided opportunities for testing the clarity of the instructions and of the job descriptions in the local languages.

**TABLE 2.** Illustrative Pair of Job Profiles Presented to CHWs During the Discrete Choice Experiment

Attributes	Job A	Job B
Supervision	Monthly CHW meetings at health center	Monthly CHW meetings at health center + quarterly visit by health center staff in the community
Training	5-day initial training and 3-day supervised practicum at health center + 3-day refresher training once a year	5-day initial training and 3-day supervised practicum at health center
Transport refund	5,000 UGX per meeting	10,000 UGX per meeting
Start-up package	CHW kit with gumboots, raincoat, job aids, and stationery + T-shirt + badge + bicycle	CHW kit with gumboots, raincoat, job aids, and stationery + T-shirt + badge
Communication	Mobile phone without airtime	No mobile phone

Abbreviations: CHW, community health worker; UGX, Ugandan shilling.

DCE data obtained from survey and IDI participants were then analyzed separately using different approaches. Choice data from the survey administration of the DCE were analyzed using mixed logit models; corresponding results are presented in the previously published article.^19^ IDIs were recorded, then transcribed directly from the recording into English. The transcripts were uploaded to NVivo for analysis. First, we compared the distribution of responses to the DCE choice tasks in the IDI group with that from the survey to assess whether the two approaches, administered to random subsets of the same CHW population, produced similar results in terms of the jobs being selected or whether asking DCE questions in a qualitative interview altered responses. Second, we used a matrix in Excel (Microsoft, Redmond, WA) to produce frequency counts of the references CHWs made to each attribute as part of the rationale for selecting jobs expressed in IDI narratives for comparison with relative importance rankings from the quantitative analysis of DCE results in the survey group. Because not all possible levels of the “start-up package” attribute were featured in each pair, frequency counts were produced at the CHW level, as opposed to the pair level. Third, sections of the IDI transcripts corresponding to the DCE were isolated and coded for observations on DCE content and on the DCE method. The content code was examined separately for the dimensions of each attribute that CHWs highlighted when (1) contrasting levels of a single attribute, and (2) when comparing jobs across attributes (i.e., trade-offs). The method code was examined inductively in a memo, looking for common sub-themes.

## RESULTS

Table 3 shows the number of CHWs who participated in an IDI from each family planning program, along with their characteristics. Most participants were women and married. CHWs in the public and former NGO programs had more experience and were more educated than CHWs in the NGO program; the NGO program was the only one in which all IDI participants did not offer injectable contraceptives.

**TABLE 3.** Number and Characteristics of CHWs Participating in a Discrete Choice Experiment via In-Depth Interview, by Type of Family Planning Program

	Public (n = 13)	NGO (n = 16)	Former NGO (n = 14)	Total (N = 43)
Age, mean, years	43	43	41	43
Number of living children, mean	6	5	6	6
Marital status, %				
Single	0	0	7	2
Married or cohabitating	100	69	79	81
Divorced, widowed, or separated	0	31	14	16
Gender, %				
Male	31	38	43	37
Female	69	62	57	63
Educational level, %				
Primary	23	50	21	33
Secondary or higher	77	50	79	67
Number of years of service, mean	7	5	10	7
Contraceptive methods provided, %				
Condoms only	0	6	0	2
Condoms and pills	0	25	0	9
Condoms, pills, and injectables	100	69	100	88^a^

Abbreviation: CHW, community health worker.

^a^ One CHW indicated providing pills and injectables but not condoms. The sum of CHWs reporting which contraceptive methods they provided does not total to 100% due to rounding errors.

### Does Administering the DCE in a Qualitative Interview Alter Responses?

As shown in Table 4, survey and IDI participants who were presented with the same block made similar choices overall. A block refers to a set of 3 pairs of jobs made up of different levels of the 5 attributes shown in Table 1, with CHWs being asked to choose their preferred job within each pair.

**TABLE 4.** Percentage Distribution of Survey and IDI Participant Responses to the Discrete Choice Experiment, by Block of Jobs Presented

Pairs of Jobs/Attribute-Level Combinations	Block 1		Block 2		Block 3		Block 4	
	Survey (n = 47)	IDI (n = 12)	Survey (n = 41)	IDI (n = 10)	Survey (n = 50)	IDI (n = 10)	Survey (n = 45)	IDI (n = 11)
Pair 1								
Job A	9	0	27	30	12	10	11	0
Job B	91	100	73	70	88	90	89	100
Pair 2								
Job A	81	67	15	20	81	70	27	9
Job B	19	33	85	80	19	30	73	91
Pair 3								
Job A	35	42	14	0	73	70	62	73
Job B	65	58	86	100	27	30	38	18
No response								9^a^

Abbreviation: IDI, in-depth interview.

Percentages of respondents selecting each job in the choices presented to them are reported for each sample (survey or IDI). Weighted percentages are reported for survey participants.

^a^ One IDI participant did not select an option for the third pair in this set.

The large majority of CHWs (36 of 43) emphasized the bicycle when explaining their selection. The next most frequently highlighted job attributes were an increased transport refund and provision of a mobile phone (29 and 27 CHWs highlighted these attributes, respectively). Overall, this is consistent with the analysis of survey results, in which 4 job attributes had a positive, significant influence on preferences: offering a start-up package with a T-shirt, badge, and bicycle had the largest impact, followed by providing a mobile phone, an increased transport refund, and adding a yearly refresher training.

Consistent with survey results, most CHWs in the in-depth interviews strategically considered the bicycle, transportation refund, and mobile phone when choosing their preferred job option.

With IDI data, we found that the bicycle was emphasized by most CHWs within each program; however, the transport refund and mobile phone did not receive equal attention in the job selection process across CHW programs. For example, over three-quarters of CHWs in the former NGO program discussed transportation refund and mobile phone job attributes when explaining their decision, while less than half of CHWs in the NGO program mentioned them. This lends additional support that results from administering the DCE to the IDI and survey groups were similar: in the quantitative analysis, results suggest that preferences for the T-shirt, badge, and package were fairly homogeneous across CHWs, but that preferences for other attributes were more heterogeneous (results are not shown here but are based on the comparison of standard deviation estimates with mean estimates for each attribute).

### What Rationale Did CHWs Offer to Support Their Decisions?

The DCE section of IDI narratives exposed CHWs' rationales in comparing the two jobs within each pair presented to them. This included attribute-specific arguments contrasting different levels of a same attribute across the two jobs or reflecting on the value of this particular attribute, as well as broader perspectives on the respective merits of the two bundles.

#### Attribute-Specific Arguments

Nineteen of the 43 CHWs explained that **bicycles** would facilitate their work by making it easier to visit clients, travel far for sensitizations, or go to the health center. Echoing comments from other CHWs, a 65-year-old male CHW in the public-sector program said that the bicycle was a decisive factor:

Many of the CHWs explained that bicycles would make it easier to visit clients, travel far, or go to the health center.

The Minister for transport [the bicycle], which will help me in my transport especially if I have to see my clients for home visits and when I have to go for group talks in some distant places ... the moment I saw the bicycle in job B, then all was well because this is one of the most important requirements for this CHW work.

Half of the CHWs who discussed the provision of a **mobile phone** said that they valued it as a program tool to exchange information with health center staff, or in the public-sector program, with other CHWs, or to improve efficiency by avoiding unnecessary trips to the health center. A couple of CHWs indicated that the utility of a phone would be limited due to the fact that most clients did not own one. Five CHWs who already owned a phone were nonetheless attracted by the idea of a new, hopefully better, phone, while 3 others felt it would be redundant. Two CHWs commented that not being provided with airtime was an issue, while 7 others indicated that a phone would still be helpful.

When it was discussed, the **transportation refund** was not systematically invoked as the basis for choosing a job. For instance, some CHWs picked the job with the smallest refund, but then lamented on the smaller refund in the job they had selected. Several CHWs, particularly in the NGO program, argued that 5,000 UGX was insufficient to cover transport (*boda boda* [a motorcycle taxi] hire) to the health center, or that it was barely sufficient, but would not leave them lunch money. In the other two programs, complaints by slightly less than half of the CHWs seemed to be fueled by the expectation that the refund would enable them to buy something (e.g., soap, food) for their family.

CHWs who were attracted by the addition of refresher **trainings** saw them as important to not forget what they had been taught, to better understand what they may not have grasped, to receive updates, and to interact with other CHWs and program managers.

Over half of CHWs in the entire sample commented on the importance of items clearly identifying them as CHWs, with a few indicating a preference for **ID cards** over **T-shirts** because they were more durable and credible. These comments were sometimes part of the rationale to choose a job, but sometimes only intended to show appreciation of what was included. The main reasons for wanting to be identified were differentiation from the rest of the community for increased popularity, credibility (in terms of qualifications), and legitimacy (particularly with husbands when visiting female clients).

For **supervision**, some CHWs underscored the value of community visits: these were seen as a boost to CHWs' credibility, an opportunity for joint sensitization and direct support, and a way for health center staff to witness firsthand the challenges CHWs faced. However, several CHWs felt that monthly meetings were sufficient, liked going to the health center and assisting the staff, or thought that the health center staff was busy and should not vacate the facility.

#### Trade-Offs Between Job Attributes

Almost a third of CHWs sometimes had difficulty choosing between jobs because they felt that several or all attributes were important. A 53-year-old woman said:

A number of CHWs had difficulty choosing between jobs because they felt that several or all attributes were important.

You cannot get all you need at the same time ... but why didn't they give a bicycle in job B? ... these people are just trying to play games with us ... something can buy you to choose a certain job, but as you continue, you notice something again enticing in the other job, so you get confused on which job to choose. ... It has really been difficult because these things are all important and, if given the opportunity, I would choose all of them.

The main trade-offs that CHWs discussed revolved around the bicycle vs. transportation refund and the mobile phone vs. bicycle; the CHWs primarily stressed the comparative advantage of the bicycle. Advantages of the bicycle over the refund were discussed by 12 CHWs. The advantages included that a bicycle could make up for a smaller refund and/or allow CHWs to save the refund money, that it was more durable, and that it was more helpful in fulfilling responsibilities. A 35-year-old female CHW said:

Here you can see that the money is a bit lower than that of job A, but it can still help you at home, but provided I have the bicycle, even if you don't give me money, then I will [be] comfortable because I can use this bicycle to transport my produce to the market from which I can earn some money ... I have been telling you that the major problem I have is that of transport, so when I saw the bicycle I immediately chose that.

Similarly, 6 CHWs highlighted the greater practical value of the bicycle over the mobile phone, such as this 41-year-old man:

I also like the bicycle and I feel it is much better than a phone ... a phone cannot make it easy for me to reach the health center to get medicine, and yet a bicycle can. I will not just call and ask them to send me the medicine.

### What Were CHWs' Experiences With the DCE?

Comments analyzed under the DCE method code shed light onto CHWs' experiences with the choice tasks. A little under a third of IDI participants commented on the jobs presented to them in a pair being “similar,” which in several cases they went on to explain meant that the difference between them was “very small.” For instance, a 39-year-old female CHW said of the two jobs presented to her:

[Choosing is] not difficult but [the jobs] are similar; the difference is very minimal.

Slightly less than one-third of the interview participants commented that the job options presented to them were similar.

Several CHWs expressed some difficulties with grasping the content of each job, at least initially, and/or said they needed time to absorb and think before choosing. A number of CHWs commented on features that were in fact present in the two jobs between which they were choosing, particularly with regard to training and supervision. This did not necessarily occur while describing a deciding factor, but rather while commenting on a job scenario and expressing appreciation for some of its features. However, a detailed examination of IDI narratives also highlighted a few inconsistencies between the features CHWs invoked in their rationales and the actual jobs presented to them, again mostly in relation to the training and supervision options (e.g., attributing refresher trainings to the wrong job in the pair). It is not entirely clear whether those inconsistencies stemmed from (1) a failure to simultaneously process all 5 attributes, (2) the nature of training and supervision options, or (3) the fact that supervision is sometimes thought as a form of refresher training, leading to possible translation errors or misinterpretation of some of the comments in the transcripts.

In a few cases, CHWs appeared to have difficulties abstracting their responses from their actual experiences: for instance, they may consider something not to be feasible. In such situations, they typically, but not systematically, were reminded by the interviewer that the scenarios were hypothetical. In at least a few cases, there was also some indication that CHWs had difficulty letting go of the jobs in the previous pair when presented with a new pair, as some of them, for instance, attempted to link jobs in the new pair to those in the previous pair.

## DISCUSSION

Although their use in public health is rapidly spreading, DCEs were originally applied in the marketing sector in high-income countries and remain a fairly novel and unfamiliar approach for such populations as CHWs. In this article, we examined qualitative data on the process of the DCE to assess whether a DCE eliciting CHWs' incentive preferences could be an appropriate and valid approach for identifying resource allocation priorities for the design of incentive packages in CHW programs. Overall, the findings of this investigation bear out the use of the DCE methodology in our context. IDI narratives highlighted the fact that participants did consider trade-offs when selecting their preferred job alternative. While the complex nature of these decisions may be obvious, this point is noteworthy as a distinctive trait, and advantage, of the DCE approach. At the same time, our experience brings attention to important design and implementation challenges from which some lessons can be derived. While these lessons overlap with guidance on how to design DCEs,^15^^,^^21^ we believe that concrete examples from the field, particularly in a context such as ours that extends the application of a DCE for health workforce issues to a different, less-educated population, are important.

**Keep the number of attributes small:** For results to be valid, participants need to be able to consider all attributes and make trade-offs when choosing between jobs in a choice pair. The number of attributes and their levels also have implications for the number of pairs that will need to be presented to each participant, which can induce fatigue. DCEs on health workforce recruitment and retention in low- and middle-income countries have used between 5 and 8 attributes, and 12 to 18 choice pairs per respondent.^21^ With CHWs, we used 5 attributes and 3 choice pairs. These numbers may be conservative because the DCE was implemented as part of either a larger survey questionnaire or a longer IDI; however, our qualitative data showed some signs that the choices may present some level of cognitive difficulty for some and required appropriate pacing and careful instructions to ensure proper understanding. Moreover, while CHWs have lower educational levels relative to formal health workers, it should be noted that all the CHWs in the combined survey and IDI sample had attended primary school, with 74% also having attended secondary school. Issues of comprehension may warrant additional attention for implementation of a DCE with CHW populations with lower education.

**Choose attribute levels that are realistic yet show sufficient range:** In our DCE, the base level for each attribute represented what CHWs typically received when the study was developed, whereas the improved levels were identified with stakeholders based on what they might realistically be willing and able to implement. While distinct, the different levels sometimes only represented what CHWs considered to be a limited range, which in turn presented some challenges. First, while it may have made it relatively easy for CHWs to envision these hypotheticals, it may also have amplified the potential for confusion with some participants commenting on the jobs being similar. Second, it is possible that the addition of the bicycle to the start-up package may in fact have been too valuable compared with the difference between the lower and higher levels of other options. While our data still show evidence of trade-offs, this may have limited our ability to obtain information on the utility of other attributes. Generally, utility balance should be considered when choosing attributes and their levels.^15^ Third, in light of the available literature and the broader study findings,^19^ we were somewhat surprised that the transport refund did not rank higher in the DCE results. Money was an important theme in the broader IDIs, although CHWs' rationales were complex and combined actual transport and opportunity costs with the desire for compensation. It appears that, while money remains an important factor, the hypothetical increase in the amount of the transport refund that stakeholders were willing to support may not have been sufficient to sway CHWs' overall choices. However, it is important to interpret DCE findings in the context of the specific options that are offered and acknowledge that money may remain a point of contention and a potential source of dissatisfaction.

While money remains an important factor to CHWs, the hypothetical increase in the amount of the transportation refund presented in the discrete choice experiment may not have been sufficient to sway the CHWs' overall choices.

**Clearly define attributes and their levels:** We found there was at times some confusion between training and supervision options. Several factors may explain this. One is that while we (and program stakeholders) considered the two to be different, the line between training and supervision was blurrier for CHWs, particularly when it came to refresher trainings and supervisory meetings. Thus, specific terms should be applied carefully when specifying attributes to avoid ambiguity. Another contributing factor may have been the fact that the levels for these two attributes were stated in increments (e.g., initial training only compared to initial training and refresher training), as opposed to mutually exclusive options (e.g., training compared to no training) that may have been easier to contrast. These issues did not transpire during the pretest we conducted for all data collection instruments, including the DCE, with a small, separate sample of CHWs; however, they may have been avoided with more extensive qualitative pilot work. Due to time and budget constraints, cross-sectional data collection with the DCE design being informed by expert knowledge and the literature was the only feasible option for this study. Preliminary qualitative work should be considered, particularly if the DCE is the only component being carried out.

The line between training and supervision may be somewhat ambiguous for CHWs, leading to some confusion between these job attributes in the discrete choice experiment.

### Limitations

The DCE was one of many components of the IDIs, thereby placing some constraints on the amount of information that could be collected in order to keep the interviews manageable. The limited qualitative data do not allow for more than a cursory examination of CHWs' decision-making processes throughout the implementation of the DCE and of their experiences with the exercise. However, they provide unique and important insights into the use of this approach with a new, low-literacy population, as compared with the health worker cadres with whom DCEs are increasingly being used to examine health workforce issues. Even though research assistants were instructed to let CHWs select their preferred job prior to probing for additional information, the process of administering the DCE qualitatively may admittedly also have introduced a bias by forcing participants to reflect on the options presented to them and to make their reasoning explicit. Nonetheless, the concordance of quantitative and qualitative results suggests that CHWs made reasoned and deliberate choices even in the absence of probing. The design of the DCE was influenced by a desire to limit the number of choice tasks submitted to participants; however, this may also have affected our ability to detect true underlying preferences. Some DCEs include a fixed choice (i.e., one pair that is the same for all the blocks) to test for internal validity. Because data from the fixed choice question are not included in mixed logit modeling and we were concerned about the total number of choice questions we could present to CHWs, this option was not retained here. However, it should be considered in future research for additional insights. Because CHWs were selected to have at least one year of experience, additional evidence may be needed to elucidate the preferences of early quitters.

## CONCLUSION

Incentive selection is a critical aspect of the design of CHW programs that tends to be informed by heuristics or by evidence on the factors associated with CHW performance and retention. Neither approach, however, is well equipped to support the prioritization of incentives, although this is an important consideration in contexts often characterized by limited resources. We found that DCEs could provide an appropriate and valid tool to obtain CHWs' incentives preferences, but that it requires careful design and implementation. Researchers and managers should consider the value of this approach for their informational needs while also being aware of its complexity. To fully appreciate the usefulness of DCEs, empirical evidence is also needed to establish the predictive value of preferences stated in a hypothetical exercise for similar real-life decisions.

## Notes

**Peer Reviewed**
